# Importance of Grading Cataracts in Predicting Recovery Time and Final Visual Outcomes After Cataract Surgery

**DOI:** 10.7759/cureus.69309

**Published:** 2024-09-13

**Authors:** Sangeetha A, K. Raviraj, Kumaresan M, Priyanka G

**Affiliations:** 1 Physiology, Mahatma Gandhi Medical College & Research Institute, Puducherry, IND; 2 Anatomy, Chettinad Hospital & Research Institute, Chennai, IND; 3 Anatomy, Mahatma Gandhi Medical College & Research Institute, Puducherry, IND; 4 Physiology, Sree Balaji Medical College & Hospital, Chennai, IND

**Keywords:** anterior segment cataract, recovery time, visual acuity, visual impairment, visual outcome

## Abstract

Introduction: Cataracts have been considered as one of the major causes for reducing the vision-related quality of life and increasing the risk of comorbidities and mortality among the general population.

Aim: This study aimed to assess the importance of grading cataracts in predicting recovery time and final visual outcomes after cataract surgery.

Method: A retrospective consecutive case review of elective cataract surgeries performed in a tertiary care hospital during a three-year period from 2019 to 2021 was studied. The postoperative visual status was correlated with grading.

Results: The reports of this study implicate the fact that 16.4% of the patients had grade 1 anterior segment cataracts. Visual acuity of perfect vision (6/6P) was obtained in 24% of patients, and 41.2% of patients had grade 1 anterior segment cataract surgery on day 1 *(p<0.005)*. Perfect vision at 6/6 visual acuity was obtained in 24% of patients, and 27.5% of patients had grade 2 anterior segment cataract surgery in week 1 *(p<0.05)*.

Conclusion: The study shows that patients with grade 1 anterior segment cataract surgery had a better visual recovery time and visual outcome. The grading system of cataracts has further shown improvement in the vision care of the patients, along with showing reliability and monitoring of cataract formation.

## Introduction

Cataracts are marked as the opacity of the eye lens that transforms the vision [1]. If not managed at the appropriate period, progressive visual loss due to cataracts can ultimately result in blindness [2]. It is most commonly witnessed in adults and the elderly. Cataracts are listed as one of the leading causes of blindness in the world [3]. It is accountable for 47.8% of blindness and estimates for 17.7 million blind people as per the World Health Organization (WHO) [4]. Numerous factors may contribute to the advancement of cataracts, thereby signifying it to be of multifactorial origin. People suffering from cataracts have a high risk of mortality and developing other comorbidities with a decrease in vision-related quality of life as compared to normal people [5]. A 2010 estimate states that cataract burden accounts for almost 33% (one-third) of all individuals with visual impairment, i.e., 94 million people, and renders complete blindness in 20 million people (51%) across the world [5]. However, visual loss due to cataracts can be restored through cataract surgery in several cases [6]. Hence, treating cataracts is thereby a necessary step toward the prevention of cataract-related visual impairment. Early and periodic eye checkups may provide one with to diagnosis of the onset of cataracts [7].

The grading of cataracts is a crucial predictor of visual outcomes in patients undergoing cataract surgeries. Cataract formation and its progress can be thus easily monitored and assessed by the specialists through grading systems. It also renders expanded data for improvements in eye care. There is a wide analysis of data regarding the visual outcome of the patient after the surgery [8]. This infers that cataract density/grading is very influential in defining the visual outcome and recovery after cataract surgery [9,10]. The complexity of cataract cases and the postoperative outcomes of cataract surgery can be thus determined using these cataract grades. Characteristics like preoperative blindness, nystagmus/strabismus, and aphakia are indicators of poor visual outcomes after cataract surgery. This implies that cataract grading plays a significant role in defining the visual outcome and recovery after cataract surgery [11,12]. This may further ease the ways for clinicians to meticulously design intraoperative strategies for enhanced quality of life among the patients. 

However, obstacles to getting these proper preventative eye care and surgical treatments persist in many remote areas in developing countries. This presents a gigantic puzzle to society, especially in cases where erratic interpretations of cataracts along with a lack of standardization were witnessed [13]. Also, the types of grading systems and surgical settings employed might show up as a variable factor in the outcome of visual acuity in various countries. Thus, the present study aims to analyze the visual outcomes and recovery rates after cataract surgery depending on the grade of cataract the patients have.

## Materials and methods

Study design and setting

A retrospective analysis of medical records of a set of patients who have undergone cataract surgery at Saveetha Medical College and Hospital, a tertiary care hospital in an urban area of Chennai, Tamil Nadu, India, was conducted. The study was approved by the institutional ethical board of Saveetha Medical College & Hospital (approval number: SMC/IEC/2021/06/146).

Study population and sample size

The study population included a total sample size of 104 patients from Saveetha Medical College & Hospital. The study included retrospective data from 2019 to 2021. After taking a detailed history, visual acuity was assessed using Snellen's chart, and a pupil dilatation test was done. Further, the lens was thoroughly examined using a panoptic ophthalmoscope. Patients underwent cataract surgery after a thorough examination. All the patients, both male and female, above the age of 35-83 years with a history of cataracts who underwent phacoemulsification were included in the study with a follow-up of one month. Patients who had traumatic cataracts were not given consent and those who had intraoperative or postoperative complications were excluded. 

Cataracts may be graded by visual inspection and numerical values, but the current study was carried out to generate a less sensitive four-point grading system, modified from Lens Opacities Classification System-II (LOCS-II). Despite its limitations, this simple four-point grading scale was used to record the opalescence of the nucleus of the lens from grades 1 to 4. The grading system followed to assess the category: Nuclei clearer than anterior/posterior sections were graded as 1; Nuclei equal to the anterior/posterior sections were graded as 2; Nuclei denser than anterior/posterior sections were graded as 3; and cataracts completely opaque/brown were graded as Brunescent or grade 4. Grade 1 indicated the early detection of and minimal clouding of the lens. All the patients were graded and examined postoperatively.

## Results

The data of the present study involve reports from 2019 to 2021 and included 104 cataract surgery patients. Only 17.3% of patients were older adults (71-80 years), whereas only 2.9% of the study population were more than 80 years old (Table 1). The frequency distribution of preoperative grading mentioned in Table 2 indicates that 20.2% of right-sided eye nuclei were equal to anterior or posterior sections. About 3.8% of the population had an opaque or brown cataract on the right side of the eye. Table 3 shows that 24.0% of the patients had perfect vision at 6/6 visual acuity (6/6P) on the first postoperative day, 24.0% of the patients had it in the first week after the operation, and 48.1% of the patients had it in the first-month follow-up. These data show visual recovery was the same on postoperative day 1 as well as week 1. The visual recovery gradually improved up to four weeks post surgery.

**Table 1:** Demographic details of the study population

Particulars	Frequency (%)
Age group (in years)	
< 40	7 (6.7%)
41-50	12 (11.5%)
51 – 60	31 (29.8%)
61 – 70	33 (31.7%)
71 – 80	18 (17.3%)
> 80	3 (2.9%)
Year of examination	
2019	30 (28.8%)
2020	22 (21.2%)
2021	52 (50%)
Year of surgery	
2019	18 (17.3%)
2020	23 (22.1%)
2021	63 (60.6%)
Type of surgery	
Multifocal	21 (20.2%)
Unifocal	83 (79.8%)

**Table 2:** Frequency distribution participants according to gades 1 to 4

	Grade 1 n(%)	Grade 2 n(%)	Grade 3 n(%)	Grade 4 n(%)
Right eye	7 (6.7 %)	21 (20.2%)	14 (13.5%)	4 (3.8%)
Left eye	9 (8.7%)	18 (17.3 %)	20 (19.2 %)	9 (8.7%)
Right and left eye	1 (1%)	1 (1%)		

**Table 3:** Vision 6/6 obtained on day 1, week 1, and month 1 6/12P: perfect vision at 6/12 visual acuity; 6/36P: perfect vision at 6/36 visual acuity; 6/6P: perfect vision at 6/6 visual acuity;  6/60P: perfect vision at 6/60 visual acuity; N6, N8, and N12 indicate results of near vision

Day 1		Week 1		Month 1	
Acuity	Freq (%)	Acuity	Freq (%)	Acuity	Freq (%)
#4/60	1 (1%)	#6/6	17 (16.3%)	#6/24P	1 (1%)
#5/60	1 (1%)	#6/12	8 (7.7%)	#6/6	17 (16.3%)
#6/12	13 (12.5%)	#6/12P	2 (1.9%)	#6/6, N6	50 (48.1%)
#6/12P	7 (6.7%)	#6/18	1 (1%)	#6/6 C strain, N6	1 (1%)
#6/18	9 (8.7%)	#6/24P	1 (1%)	#6/6P	8 (7.7%)
#6/24	3 (2.9%)	#6/6	12 (11.5%)	#6/6P, N6	13 (12.5%)
#6/36	2 (1.9%)	#6/6, N6	2 (1.9%)	#6/60	1 (1%)
#6/36P	2 (1.9%)	#6/6 (-2)	1 (1%)	#6/60, N6	1 (1%)
#6/6	7 (6.7%)	#6/6P	25 (24%)	#6/9	7 (6.7%)
#6/6 , N6	1 (1%)	#6/6P N6 P	1 (1%)	#6/9, N6	4 (3.8%)
#6/6P	25 (24%)	#6/6P N6	1 (1%)	#6/6 EST N6	1 (1%)
#6/6P N6	1 (1%)	#6/60	3 (2.9%)	Total	104 (100%)
#6/60P	1 (1%)	#6/9	22 (1%)		
#6/9	20 (19.2%)	#6/9P	6 (5.8%)		
#6/9, N8	1 (1%)	#6/9 EPH 6/6P	2 (1.9%)		
#6/9 N12	1 (1%)	Total	104 (100%)		
#6/9P	9 (8.7%)				
Total	104 (100%)				

About 41.2% of the patients who had a clear nucleus during the preoperative stage had a vision of 6/6P during postoperative day 1. None of the patients had normal visual acuity whose nuclei were brown or opaque during preoperative assessment. The acuity of vision for near vision was poor irrespective of cataract grades (p<.005). The result also showed that a very small (2.5%) population had poor visual acuity (4/60) (Table 4). Interestingly, during week 1 follow-up, about 35.3% of patients who had a clear nucleus had normal vision of 6/6P. Near vision (N6) (1%) showed poor outcome during week 1, irrespective of cataract grades. Twenty-four percent of grade 4 patients had normal 6/6P vision (p<0.05) (Table 5).

**Table 4:** Comparison between the grade of cataracts and outcomes on postoperative day 1 and anterior segment cataract grade * There is a significant difference (p<0.005) between the grade of anterior segment cataract and best-corrected vision on day 1. 6/12P: perfect vision at 6/12 visual acuity; 6/36P: perfect vision at 6/36 visual acuity; 6/6P: perfect vision at 6/6 visual acuity;  6/60P: perfect vision at 6/60 visual acuity; N6, N8, and N12 indicate results of near vision

Acuity of vision	Grade 1 (% within grade)	Grade 2 (% within grade)	Grade 3 (% within grade)	Grade 4 (% within grade)	Total	Pearson's chi-square
#4/60	0 (0.0%)	1 (2.5%)	0 (0.0%)	0 (0.0%)	1 (1.0%)	p-value .004*
#5/60	0 (0.0%)	0 (0.0%)	1 (2.9%)	0 (0.0%)	1 (1.0%	
#6/12	0 (0.0%)	3 (7.5%)	8 (23.5%)	2 (15.4%)	13 (12.5%)	
#6/12P	1 (5.9%)	3 (7.5%)	1 (2.9%)	2 (15.4%)	7 (6.7%)	
#6/18	0(0.0%)	2 (5.0%)	7 (20.6%)	0 (0.0%)	9 (8.7%)	
#6/24	0 (0.0%)	0 (0.0%)	1 (2.9%)	2 (15.4%)	3 (2.9%)	
#6/36	0 (0.0%)	0 (0.0%)	0 (0.0%)	2 (15.4%)	2 (1.9%)	
#6/36P	0 (0.0%)	0 (0.0%)	1 (2.9%)	1 (7.7%)	2 (1.9%)	
#6/6	2 (11.8%)	3 (7.5%)	1 (2.9%)	1 (7.7%)	7 (6.7%)	
#6/6, N6	0 (0.0%)	1 (2.5%)	0 (0.0%)	0 (0.0%)	1 (1.0%)	
# 6/6P	7 (41.2%)	12 (30%)	6 (17.6%)	0 (0.0%)	25 (24.0%)	
#6/6P, N6	1 (5.9%)	0 (0.0%)	0 (0.0%)	0 (0.0%)	1 (1.0%)	
#6/60P	0 (0.0%)	0 (0.0%)	0 (0.0%)	1 (7.7%)	1 (1.0%)	
#6/9	4 (23.5%)	8 (20.0%)	6 (17.6%)	2 (15.4%)	20 (19.2%)	
#6/9, N8	0 (0.0%)	1 (2.5%)	0 (0.0%)	0 (0.0%)	1 (1.0%)	
#6/9, N12	0 (0.0%)	1 (2.5%)	0 (0.0%)	0 (0.0%)	1 (1.0%)	
#6/9P	2 (11.8%)	5 (12.5%)	2 (5.9%)	0 (0.0%)	9 (8.7%)	

**Table 5:** Comparison between the grade of cataracts and outcomes after one week and anterior segment cataract grade **There was a statistically significant difference in the vision of patients with different grades of cataracts since the p<0.05. 6/12P: perfect vision at 6/12 visual acuity; 6/36P: perfect vision at 6/36 visual acuity; 6/6P: perfect vision at 6/6 visual acuity;  6/60P: perfect vision at 6/60 visual acuity; N6, N8, and N12 indicate results of near vision

Acuity of vision	Grade 1 (% within grade)	Grade 2 (% within grade)	Grade 3 (% within grade)	Grade 4 (% within grade)	Total	Pearson’s chi-square
#6/6	4 (23.5%)	8 (20.0%)	4 (11.8%)	1 (7.7%)	17 (16.3%)	0.022**
#6/12	0 (0.0%)	1 (2.5%)	7 (20.6%)	0 (0.0%)	8 (7.7%)	
#6/12P	0 (0.0%)	1 (2.5%)	0 (0.0%)	1 (7.7%)	2 (1.9%)	
#6/18	0 (0.0%)	0 (0.0%)	0 (0.0%)	1 (7.7%)	1 (1.0%)	
#6/24P	0 (0.0%)	0 (0.0%)	0 (0.0%)	1 (7.7%)	1 (1.0%)	
#6/6	3 (17.6%)	8 (20.0%)	1 (2.9%)	0 (0.0%)	12 (11.5%)	
#6/6, N6	1 (5.9%)	1 (2.5%)	0 (0.0%)	0 (0.0%)	2 (1.9%)	
#6/6 ( -2 )	0 (0.0%)	0 (0.0%)	1 (2.9%)	0 (0.0%)	1 (1.0%)	
#6/6P	6 (35.3%)	11 (27.5%)	7(20.6%)	1 (7.7%)	25 (24.0%)	
#6/6P, N6 P	0 (0.0%)	1 (2.5%)	0 (0.0%)	0 (0.0%)	1 (1.0%)	
#6/6P, N6	0 (0.0%)	1 (2.5%)	0 (0.0%)	0 (0.0%)	1 (1.0%)	
#6/60	0 (0.0%)	1 (2.5%)	1 (2.9%)	1 (7.7%)	3 (2.9%)	
#6/9	1 (5.9%)	6 (15.0%)	9 (26.5%)	6 (46.2%)	22 (21.2%)	
#6/9P	2 (11.8%)	1 (2.5%)	3 (8.8%)	0 (0.0%)	6 (5.8%)	
#6/9 EPH 6/6P	0 (0.0%)	0 (0.0%)	1 (2.9%)	1 (7.7%)	2 (1.9%)	
Total	17 (100.0%)	40 (100.0%)	34 (100.0%)	13 (100.0%)	104 (100.0%)	

## Discussion

The prediction of final visual outcomes after cataract surgery has been known to be a challenging approach among patients. The patients who are mainly suffering from the concomitant retinal disease are prone to risk with the unsuccessful outcome, and there is a need for implicating accurate prediction of visual acuity within the population. Hence, different procedures have been integrated for assessing the final visual outcomes after cataract surgery along with the prediction of recovery time among the patients undergoing cataract surgery [14, 15]. The use of the Mentor Guyton-Minkowski potential acuity meter (PAM) and machine-based potential vision tests are not available to the ophthalmologists, mainly in developing countries [16]. Another tool, Lens Opacities Classification System III (LOCS III), a grading system, is currently used both clinically and for research purposes [17, 18]. Due to their complexity and postoperative outcome, there was a need for a newer and simpler tool. Hence, the present study has aimed for evaluating the visual outcome and recovery after cataract surgery depending on the grade of the cataract (grade 1 to grade 4). 

However, there was a high statistical significance on visual recovery on day 1 with grade 1 anterior segment cataract surgery. The possible reason could be due to less energy expenditure during phacoemulsification and less inflammation after surgery [19]. Cataract patients who live in urban areas had faster recovery due to the availability of eye care [20]. However, elderly people aged more than 60 had complete recovery after four to 16 weeks of surgery [21]. Both phaco and extra capsular cataract extraction (ECCE) with an intraocular lens (IOL) in very elderly patients were found to be safe if the systemic conditions were stable, and the literature also emphasizes that advanced age was not a contraindication for surgery [22]. Most of the patients who underwent immediate sequential bilateral cataract surgery (ISBCS) demonstrated meaningful improvement in uncorrected distance visual acuity (UDVA) compared with preoperative corrected distance visual acuity (CDVA) as early as postoperative day 1. [23] Another study from London, UK, on small incision surgery with phacoemulsification showed significantly fewer complaints and higher visual recovery. [24]

Modern cataract surgery is safe in more than 95% of patients. However, knowledge of the postoperative complaints associated with ophthalmic surgery is necessary because these complaints may delay recovery and may significantly influence quality of life after the procedure. Pain after ophthalmic surgery is often considered to be a sign of a possible complication [25]. The most important preoperative step is to assess high-risk individuals and counsel them appropriately. For such patients, various steps on postoperative management can be taken in addition to routine phaco surgery to optimize their visual outcome and improve quality of life.

Limitations

Only the nucleus of the lens was included in the grading system, while other important factors like subcapsular opacities, interpupillary space, and posterior capsular area were not taken into consideration. Photographic references of the nucleus of the lens were not included as the study focused only on the lens opalescence of the nucleus. It has been acknowledged that the anticipated outcomes of cataracts and cataract surgery may serve to deviate based upon the geographic location, study population, and center facilities. The study was designed and carried out at a tertiary care hospital in an urban area in Chennai, India. It is a single-center study, which might add to the limitations of the available data. Being a retrospective study, it was difficult to find the impact of the COVID-19 pandemic and diabetes in the study population.

## Conclusions

In conclusion, this study found an improvement in the subjective quality of vision after cataract surgery in patients undergoing cataract surgery. This also proves that patients with grade 1 anterior segment cataract surgery had a better visual recovery time and visual outcome compared to grades 2, 3, and 4. Hence, the above data have justified the cataract grading, which is an important predictor of visual outcomes among patients undergoing cataract surgery. The grading system of cataracts has further shown improvement in the vision care of the patients, along with showing reliability and monitoring of cataract formation.

## References

[1] Liu YC, Wilkins M, Kim T, Malyugin B, Mehta JS (2017). Cataracts. Lancet.

[2] Pascolini D, Mariotti SP (2012). Global estimates of visual impairment: 2010. Br J Ophthalmol.

[3] Song P, Wang H, Theodoratou E, Chan KY, Rudan I (2018). The national and subnational prevalence of cataract and cataract blindness in China: a systematic review and meta-analysis. J Glob Health.

[4] Rao GN, Khanna R, Payal A (2011). The global burden of cataract. Curr Opin Ophthalmol.

[5] Song E, Sun H, Xu Y, Ma Y, Zhu H, Pan CW (2014). Age-related cataract, cataract surgery and subsequent mortality: a systematic review and meta-analysis. PLoS One.

[6] Barraquer RI, Pinilla Cortés L, Allende MJ (2017). Validation of the nuclear cataract grading system BCN 10. Ophthalmic Res.

[7] Klein BE, Klein R (2013). Projected prevalences of age-related eye diseases. Invest Ophthalmol Vis Sci.

[8] Gali HE, Sella R, Afshari NA (2019). Cataract grading systems: a review of past and present. Curr Opin Ophthalmol.

[9] Thylefors B, Chylack LT Jr, Konyama K, Sasaki K, Sperduto R, Taylor HR, West S (2002). A simplified cataract grading system. Ophthalmic Epidemiol.

[10] Bullimore MA, Bailey IL (1993). Considerations in the subjective assessment of cataract. Optom Vis Sci.

[11] Sorrentino FS, Matteini S, Imburgia A, Bonifazzi C, Sebastiani A, Parmeggiani F (2017). Torsional phacoemulsification: a pilot study to revise the "harm scale" evaluating the endothelial damage and the visual acuity after cataract surgery. PLoS One.

[12] Mndeme FG, Mmbaga BT, Msina M (2021). Presentation, surgery and 1-year outcomes of childhood cataract surgery in Tanzania. Br J Ophthalmol.

[13] Khanna R, Pujari S, Sangwan V (2011). Cataract surgery in developing countries. Curr Opin Ophthalmol.

[14] Westborg I, Mönestam E (2020). Follow-up after cataract surgery - comparison of the practice in two institutions with the aim of optimize the routine. Clin Ophthalmol.

[15] Macky TA, Mohamed AM, Emarah AM, Osman AA, Gado AS (2015). Predicting postoperative visual outcomes in cataract patients with maculopathy. Indian J Ophthalmol.

[16] Minkowski JS, Palese M, Guyton DL (1983). Potential acuity meter using a minute aerial pinhole aperture. Ophthalmology.

[17] Wong WL, Li X, Li J (2013). Cataract conversion assessment using lens opacity classification system III and Wisconsin cataract grading system. Invest Ophthalmol Vis Sci.

[18] Cuzzani OE, Ellant JP, Young PW, Gimbel HV, Rydz M (1998). Potential acuity meter versus scanning laser ophthalmoscope to predict visual acuity in cataract patients. J Cataract Refract Surg.

[19] Beyene AM, Eshetie A, Tadesse Y, Getnet MG (2021). Time to recovery from cataract and its predictors among eye cataract patients treated with cataract surgery: a retrospective cohort study in Ethiopia. Ann Med Surg (Lond).

[20] Rabiu MM (2001). Cataract blindness and barriers to uptake of cataract surgery in a rural community of northern Nigeria. Br J Ophthalmol.

[21] Alió J, Rodríguez-Prats JL, Galal A, Ramzy M (2005). Outcomes of microincision cataract surgery versus coaxial phacoemulsification. Ophthalmology.

[22] Michalska-Małecka K, Nowak M, Gościniewicz P, Karpe J, Słowińska-Łożyńska L, Łypaczewska A, Romaniuk D (2013). Results of cataract surgery in the very elderly population. Clin Interv Aging.

[23] Kwedar K, Arnold J, Hesemann N (2022). Visual recovery after immediate sequential bilateral cataract surgery at a veterans' hospital. J Cataract Refract Surg.

[24] Minassian DC, Rosen P, Dart JK (2001). Extracapsular cataract extraction compared with small incision surgery by phacoemulsification: a randomised trial. Br J Ophthalmol.

[25] Porela-Tiihonen S, Kokki H, Kaarniranta K, Kokki M (2016). Recovery after cataract surgery. Acta Ophthalmol.

